# Facile construction of calcium titanate-loaded silk fibroin scaffolds hybrid frameworks for accelerating neuronal cell growth in peripheral nerve regeneration

**DOI:** 10.1016/j.heliyon.2023.e15074

**Published:** 2023-04-01

**Authors:** Jincheng Zhang, Yiwen Xu, Yingqi Zhang, Lei Chen, Yeqing Sun, Jia Liu, Zhitao Rao

**Affiliations:** a Department of Sports Medicine, Tongji Hospital, School of Medicine, Tongji University, 389, Xincun Road, Putuo, Shanghai 200065, PR China; b Department of Stomatology, Shanghai Tenth People's Hospital, School of Medicine, Tongji University, No. 301 Yanchang Road, Shanghai 200072, China; c Department of Orthopedics, Tongji Hospital, School of Medicine, Tongji University, 389 Xincun Road, Putuo, Shanghai 200065, PR China

**Keywords:** Silk fibroin, Calcium titanate, Cell proliferation, Peripheral nerve regeneration

## Abstract

Different concentrations of calcium titanate (CaTiO3) nanoparticles were loaded into the Silk fibroin (SF) solution to construct porous SF@CaTiO3 hybrid scaffolds, which were shown to have enhanced properties for stimulating peripheral nerve regeneration. Surface charges, crystallization intensity, wettability, porosity, and morphology were measured and analyzed. We analyzed the hybrid porous SF@CaTiO_3_ scaffolds that affected the expansion of Schwann cells. The results demonstrated a concentration-dependent influence on the dispersion of nanoparticles in the CaTiO_3_ hybridized SF scaffolds. Incorporating CaTiO_3_-NPs into the porous SF@CaTiO_3_ hybrid scaffolds can boost hydrophobicity while decreasing surface charge density and porosity. The hybridized scaffolds mostly had an orthorhombic calcium titanate crystal structure with amorphous Silk fibroin mixed. Schwann cell cultures revealed that SF@CaTiO_3_ hybrid scaffolds containing an optimal CaTiO_3_-NPs concentration could stimulate the proliferation, attachment, and protection of Schwann cell biological functions, suggesting the scaffolds' potential for use in peripheral nerve regeneration.

## 1 Introduction

Muscle weakening, neurotropic ulceration, and a diminished or lost touch sensation are only some long-term effects that can arise from a peripheral nerve injury (PNI), which is a terrible condition in and of itself [1,2,3]. Traumatic injuries, electrical injuries, metabolic disorders, infections, and drug injection injuries can all lead to various PNIs, including stretch-related injuries (traction), lacerations, compression, mechanical deformation, and severe bruising (contusion) [4]. Regarding treating PNI, autologous nerve transplantation is currently the best option [5]. The restricted supply of the donor's nerves, the morbidity of the donor site, the painful growth of neuromas, the risk of infection at the donor site, and the incompatibility of the donor nerve and the recipient site all negatively impact clinical outcomes despite the method's success [6].

An unprecedented number of efforts have been made in recent years to propose new methods of repairing PNI using well-built biomaterials [7]. Biomaterials like nanofibers, nanostructured materials, and hydrogels, each with unique morphology and physicochemical properties, have all been proposed and examined as potential treatments for injured neuron regeneration [8]. Natural polymer-based hydrogels have attracted much interest as a type of biomaterial because of their unique features, including their excellent biological and physicochemical capabilities [9]. Hydrogels' stiffening and viscoelasticity can be easily adjusted to mimic the natural nerve's mechanical properties [10]. And hydrogels' expanded 3D design can sustain and speed up neuronal development. Clinical investigations have shown that biodegradable nerve guides effectively replace native nerve grafts in treating relatively short nerve deficits [11]. The guide's capacity to lead the developing nerve fibers towards the distal nerve stump and avoid the development of a neuroma by blocking the ingrowth of fibrous tissue are both benefits of this treatment method [12,13,14]. They identified a guide with no cellular toxicity, high tensile strength, and a low degradation rate for effective peripheral nerve healing.

Silk fibroin, obtained from the cocoons of the *Bombyx mori* silkworm, has been extensively studied for its potential medical uses [15]. Studies have demonstrated that silk protein has low cytotoxicity, is biocompatible, has minimal swelling, high tensile strength, and high breaking strength. Studies show that histomorphometric metrics were used to evaluate neural regeneration in tissue slices, including fiber and axon areas, g-ratio, fiber density, and myelin sheath thickness [16]. As an additional measure, the gastrocnemius weight ratio was used to evaluate functional recovery after nerve gap injury [17]. A collagen type I guide approved by the Food and Drug Administration was utilized to evaluate the silk nerve guide [18]. Using collagen tubulization to close nerve gaps shorter than 10 mm in length has shown equivalent neuroregenerative performance to nerve autografts in mouse and nonhuman monkey experimental models [19]. This study also included an autograft group as a positive control [20]. Silk's rich output, superior mechanical features, color sense shine, and soft and sensitive feel have earned it the title of “Queen of Fibers,” As a natural green protein substance that has garnered considerable attention in biomedicine, silk fiber's functional characteristics and practical uses [21,22,23]. The silk fiber is an important component of biological products like chitosan, polylactic acid, and collagen. Creating mulberry silkworms, spinning the silk into cocoons, and mechanically removing the raw silk from the cocoons are all steps in acquiring natural silk fiber. Due to its better biocompatibility, high permeability, nontoxicity, nonirritating, and mechanical properties, silk fibroin has become a center of strong contemporary study [24]. Researchers in the biomedical field have found silk protein hydrogels to be the most promising of the many silk fiber-based biomaterials due to their superb biological characteristics, internal structures such as extracellular matrices, and diverse gel techniques. However, the processes of their three-dimensional structure and functional units of biological activity, as well as fundamental research on and practical uses of silk strands, are still in their infancy [25]. Consistent, in-depth study of silk fibers has allowed for the slow resolution of connected issues. It is the hastening study of novel products, biological activity, and useful materials based on silk fibers. Investigation into silk protein hydrogels' biocompatibility, durability, alternative mechanical characteristics, and drug-carrying systems will be crucial to expanding the treatment's reach while decreasing its financial and environmental toll on the human body [26,27,28].

Perovskite-structured metal titanate compounds, including calcium titanate (CaTiO_3_), interest basic and applied researchers. It has been studied extensively for its structural, electric, and optical properties because of its versatility in structural transformations and applications in ferroelectric and dielectric ceramic materials, high-performance capacitors, sensors, and electroluminescent devices [29]. Due to its negative temperature coefficient is also known as an impressively chemically resistant n-type semiconductor, a ceramic dielectric with a high dielectric constant, and a thermal resistive element [30]. Nano- and micron-sized CaTiO_3_ particles, films, and composites have also received significant research and development attention. Due to their high surface areas and small sizes, these species are used in various applications, including but not limited to environmental remediation; electronics; catalytic and photocatalytic processes; biomimetic calcium phosphates production; biomedical engineering [31,32].

This investigation combined Silk fibroin and CaTiO_3_-NPs to create porous hybrid biomaterials. The properties of the SF@CaTiO_3_ porous scaffolds were analyzed, including their porosity, wettability, surface charges, crystal intensity, and microstructure. Schwann cells were cultured to evaluate peripheral nerve regeneration potential in vitro. Schwann cells were cultured on scaffolds and analyzed for their shape, proliferation, and protrusions. Porous Silk fibroin scaffolds were studied in their purified form and after processing.

## 2 Experimental section

### 2.1 Reagents and materials

The other compounds used in this work were of analytical grade (AR) and were used without further purification. Tussah cocoons were obtained from Nanyang City in Henan Province, China. Cocoons were degummed thrice at 98 °C for 30 min using 0.5 wt% Na_2_CO_3_. Degummed tussah fibers were dissolved by diluting 35-fold in 9 M aqueous lithium thiocyanate and stirring at 55 °C for 1 h. Subsequently, the solution was filtered to remove the remaining particulates and dialyzed for 3 days against distilled water. A 31 wt% aqueous solution was prepared by dialysis in 35 wt% PEG according to published methods [33,34]. CaTiO_3_ nanoparticles (98%) were purchased from Shanghai Aladdin Biochemical Technology Co., Ltd. (Shanghai, China). Ethanol4% formaldehyde and acetic acid were obtained from Sinopharm (Shanghai Sinopharm Group Chemical Reagent Co., Ltd.). Cell counting kit-8 (CCK-8) Toluidine Blue O (TBO) were purchased from was purchased from TCI (Shanghai) Development Co., Ltd. (Shanghai, China). Phosphate-buffered saline streptomycin and penicillin (PBS, pH 7.3) were obtained from Beyotime Biotechnology Co., Ltd. (Shanghai, China). Ultrapure water (18.2 MΩ cm^−1^) applied in all experiments was produced by a Milli-Q purification system. Fetal bovine serum (FBS), trypsin, and Dulbecco's Minimum Essential Medium (DMEM) were purchased from Beijing Solarbio Science & Technology Co., Ltd. Brain-derived neurotrophic factor (BDNF) kit, ciliary neurotrophic factor (CNTF) kit. The nerve growth factor (NGF) kit was supplied by Solarbio Technology Co., Ltd. (Beijing, China).

### 2.2 Characterisation of nanoparticles

The surface zeta potential (ζ) of CaTiO_3_-NPs was established at 25 ± 2 °C using disposable plain-folded capillary zeta cells on a Zetasizer ZS90 (Malvern Instruments, Malvern, UK). CaTiO_3_-NPs were diluted (10 times) with deionized water before size, and each CaTiO_3_-NPs was analyzed in triplicate. Scanning electron microscopy (SEM) images of CaTiO_3_-NPs were obtained with a JSM-7500 field emission scanning electron microscope. The crystalline phase of the CaTiO_3_-NPs was analyzed by X-ray diffraction (Rigaku D/Max 2550).

### 2.3 Preparation of aqueous tussah silk fibroin (SF) and SF@CaTiO_3_ hybrid scaffolds

Cocoons were degummed thrice using 0.5 wt% Na_2_CO_3_ at 98 °C for 30 min. Tussah fibers that had been degummed were dissolved after diluting 35 times in 9 M aqueous lithium thiocyanate and then stirred at 55 °C for an hour. The solution was filtered to eliminate any leftover particles before being dialyzed for three days against distilled water. Dialysis in 35 wt% PEG was used to create a 31 wt% aqueous solution under established procedures.

**Fig. 1:**
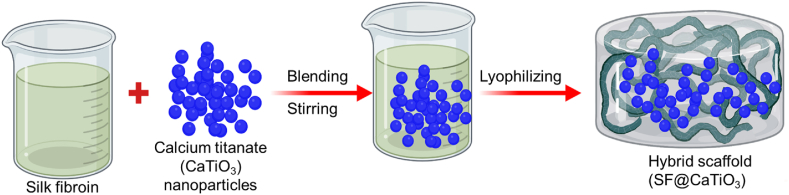
Graphical representation of silk fibroin (SF) fabrication with calcium titanate (CaTiO_3_) hybrid scaffolds for peripheral regeneration.

The 1% Silk fibroin solution was constructed by dissolving 1 g of Silk fibroin in 1% (v/v) acetic acid solution and stirring the mixture for 3-h at room temperature (RT). We then stood the solutions at RT for 24-h to force out any remaining air bubbles. Then, 1 mg, 5 mg, and 10 mg of CaTiO_3_-NPs were introduced to a 10 mL SF solution and thoroughly blended. Strong stirring was followed by degassing the solutions in a vacuum oven. A 24-well culture plate had 0.5 mL of the solution applied to each well, and the plate was lyophilized for 24-h at 55 °C. The newly fabricated scaffolds were soaked and peeled off in a NaOH (4%) solution for 5-h to neutralize any lingering AA. Ultimately, the scaffolds were neutralized by being carefully rinsed with DH_2_O. The lyophilized hybrid scaffolds were numbered: SF@CaTiO_3_-1, SF@CaTiO_3_-5, and SF@CaTiO_3_-10. Additionally, SF scaffolds devoid of CaTiO_3_ were generated using the same procedure and served as a control (Fig. 1).

### 2.4 Porosity of nanoparticles

According to the literature and with certain adjustments made in this investigation [30], the hybridized scaffolds with known weight (Wk) were soaked in ethanol for at least 10 min under vacuum conditions (0.08 MPa) to remove the air bubbles. After that, the hybridized scaffolds were removed, and the excess ethanol was absorbed rapidly using filter paper. Immediately, the scaffolds were weighed again and recorded as wet weight (Ww). Finally, the porosity (P) of the hybridized scaffolds was defined in terms of the following formula:

P=(Ww−Wk)/(ρVs)×100%

Herein, ρ was the density of ethanol (0.789 mg/mL) at room temperature. Vs. could be obtained from the geometry of the scaffolds according to the diameter and height of the cross-section.

### 2.5 Measurement of water contact angle (WCA) of nanoparticles

The surface wettability of the CaTiO3 hybridized porous Silk fibroin scaffolds was studied by measuring contact angles using a sessile drop method with DH_2_O deposited on the scaffold surface. The measurement was performed using a contact angle measurement instrument (JY-PHa, Chengde): a drop of DH2O was first added onto the surface of the scaffold with the size of 10 × 10 mm. Then, the drop was stabilized for 3 s and recorded using an inspection microscope and digital imaging techniques. The images were processed using the software embedded in the instrument for obtaining contact angle values. Three parallel samples were used for each measurement [35,36,37].

### 2.6 Charge measurement of nanoparticles

The surface charges of the fabricated hybridized scaffolds were measured using an EST111 Static Charge Meter (EST Electro-Static Test, Co. Ltd., China) in the following ways: Hybridized scaffolds with a 10 mm diameter were put in the measuring chamber, and the resulting data was collected after the display had stabilized. Calculating at least three measurements for each scaffold determined the average surface charges.

### 2.7 Cell culture

Schwann cells were collected and cultivated on experiments using the subsequent method: The sciatic nerves were removed from 2 to 5-day-old S-D rat pups and enzymatically processed with 0.125% trypsin and 1% collagenase were maintained in plastic dishes at 37 °C under a humidified atmosphere of 5% CO_2_. Then, the mixtures were resuspended and centrifuged in DMEM with 10% FBS, and the cell suspensions were added to the Petri dish. After 24 h, the Schwann cells were distilled by adding 10 mM cytosine arabinoside to the above dishes and incubated for 48 h to remove fibroblasts. Subsequently, 2 mM forskolin and 2 ng/mL neuregulin were used to stimulate cell proliferation [38,39,40,41,42]. Later, the cells were incubated at 37 °C under a humidified atmosphere of 5% CO_2_ before use.

#### 2.7.1 Examination of CCK-8 analysis

Cell viability was examined by the typical Cell Counting Kit-8 (CCK-8) assay. Cells were plated in 96-well plates (1 × 10^4^ cells per well) and cultivated for 24 h. After eliminating the culture medium, 200 μL of DMEM comprising different concentrations of different samples was added. After various days of treatment, the cells were incubated. After the solution was rinsed by PBS twice, 100 μL of DMEM, including 10 μL of CCK-8 solution, was added, and the cells were further incubated for 1.5 h. The absorbance recorded the cell viability at 450 nm on a Tecan Spark microplate reader (Tecan, Mannedorf, Switzerland).

#### 2.7.2 TBO staining of nanoparticles

Using a TBO experiment to investigate the Schwann cells were distributed throughout the SF@CaTiO_3_ porous hybrid scaffolds. These scaffolds were washed thrice with PBS to eliminate any remaining DMEM media after 1, 3, and 5 days of cell growth. After allowing each well to rest for at least 2-h in PBS, 1 mL of newly fabricated 4% formaldehyde was added. After letting the scaffolds in each well respond for 30 min in PBS containing 1 wt% TBO, the scaffolds were washed three times with PBS. Using a microscope, the images were taken from the CKX53 Olympus microscope (Tokyo, Japan).

#### 2.7.3 Investigation of cell protrusion

Using Image Pro Plus, we collected and quantified Schwann cell projections of varying lengths and densities. Schwann cells were stained with TBO according to the procedure after being fixed in 4% formaldehyde. The cells were examined and imaged using a microscope and a portable CCD camera. The protrusion outgrowth may be studied in detail by measuring the number of cells. Image Pro Plus also allowed for accurate measurement of the length of each somatic protrusion.

### 2.8 Schwann cells growth factors

Schwann cells' biological activity was measured by detecting the release of brain-derived neurotrophic factor (BDNF), nerve growth factor (NGF), and ciliary neurotrophic factor (CNTF) in different types of constructed scaffolds. After 1- and 3-days incubation at 37 °C, the supernatant from each well was collected as the specimen and kept at 20 °C. Following the protocol outlined in the product insert, ELISAs were used to detect the excretion of BDNF, NGF, and CNTF. BDNF, NGF, and CNTF can all be accurately measured using these assays. Their minimum detection levels of 10 pg/mL of BDNF, NGF, and CNTF are all well within previous studies' detection ranges. The concentration of the individual components was determined using the calibration curve based on the optical density measured at a wavenumber of 450 nm. In each experiment, five samples were taken in parallel.

### 2.9 Statistical analysis

All data were presented as mean ± standard deviation (SD). The experimental results were analyzed by one-way analysis of variance (ANOVA) by using GraphPad prism software. p-value of <0.05, 0.01 and 0.001 were considered a statistically significant diﬀerence and marked with *, ** and ***, respectively.

## 3 Results and discussions

### 3.1 Characterization of CaTiO_3_-NPs

**Fig. 2:**
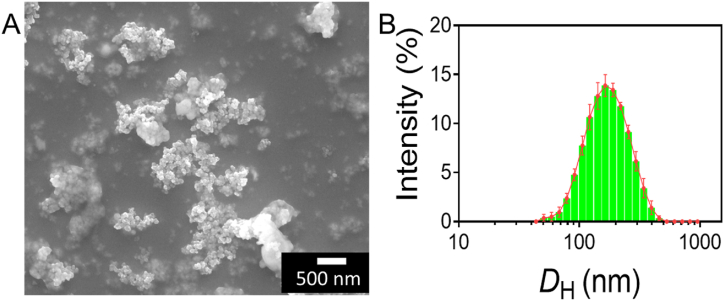
Morphological characterization of CaTiO_3_. A) Morphology of CaTiO_3_ investigated by scanning electron microscopy (SEM) analysis. B) The distribution diameter CaTiO_3_ was investigated by dynamic light scattering (DLS) analysis. Data are presented as mean ± SD. Scale bar 500 nm.

CaTiO_3_ nanoparticle (CaTiO_3_-NPs) profiles were observed, and their geometric dimensions were confirmed using a scanning electron microscope (SEM) and a hydrodynamic particle sizer. Fig. 2A displays the particle shape and size of CaTiO_3_-NPs as determined by the experiment. CaTiO_3_-NPs typically had sizes between 110 and 210 nm in diameter (Fig. 2B). Over 61% of the nanoparticles measured between 150 and 190 nm in size. The SEM findings also revealed the dispersion of CaTiO_3_-NPs. Most nanoparticles were spherical and clustered together, as was readily visible. CaTiO_3_-NPs aggregation may be mostly attributable to the extremely high concentration used in this experiment. The findings suggested that nanosized CaTiO_3_ particles predominated, which bodes well for the nano-effect of CaTiO_3'_s potential utility in future cell growth and tissue engineering. Perovskite/PCL hybrid scaffolds were created by Bagchi et al. [43]. With nanoparticle sizes between 85 and 125 nm. They incorporated nanoparticles into the composite's improved mechanical characteristics, suggesting increased efficacy in encouraging bone tissue engineering [44].

**Fig. 3:**
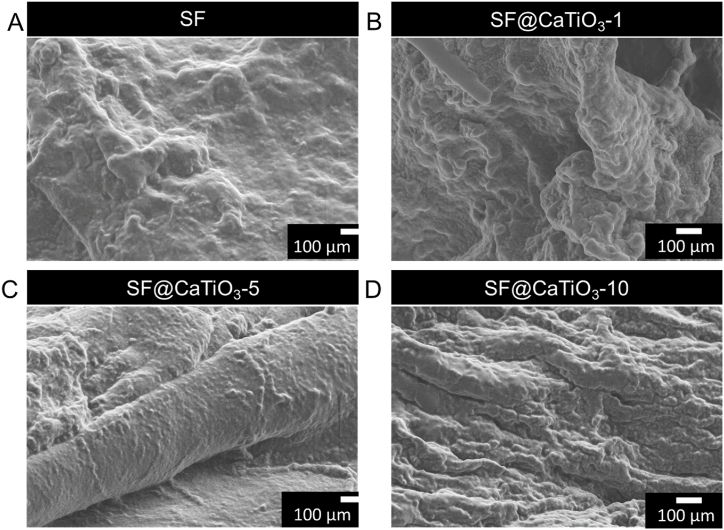
A-D) The pore size of SF scaffolds with CaTiO_3_-NPs, the different concentrations of CaTiO_3_ nanoparticles (SF, SF@CaTiO_3_-1, SF@CaTiO_3_-5, and SF@CaTiO_3_-10) were investigated by SEM analysis. Scale bar 100 μm.

A scanning electron microscope (SEM) was used to examine the SF@CaTiO_3_ hybrid scaffolds after fabrication. Fig. 3A–D shows SEM images of the hybrid scaffold at varying concentrations of CaTiO_3_. Pure SF scaffolds had a strip-like structure and were porous throughout. The scaffolds' distinctive structure nearly vanished after hybridization with CaTiO_3_. No hybridized scaffold didn't prominently display CaTiO_3_-NPs. Low concentrations of CaTiO_3_-NPs (0.1 mg/mL) resulted in a dispersed distribution of nanoparticles over the scaffolds, while higher concentrations (0.5 and 1 mg/mL) fabricated clustered nanoparticles. Additionally, CaTiO_3_-NPs in SF scaffolds were distributed uniformly, either coating the surface or being embedded within the structure. This study's findings demonstrated a concentration-dependent impact from the SF@CaTiO_3_.

**Fig. 4:**
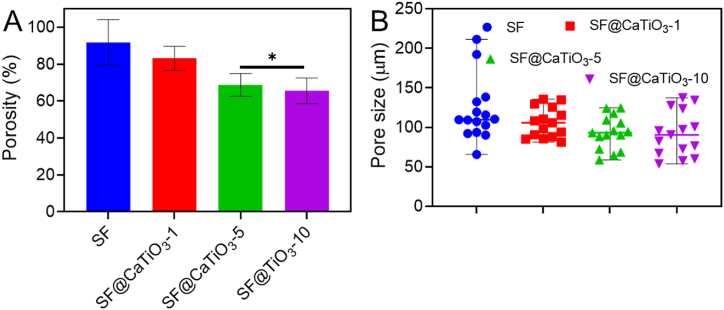
A) Porosity and B) Pore size of SF, SF@CaTiO_3_-1, SF@CaTiO_3_-5, and SF@CaTiO_3_-10. Data are presented as mean ± SD. *P < 0.05.

Fig. 4A displays the hybridized scaffolds' porosity. The porosity of the pure SF scaffold was around 82%. However, the scaffolds' porosity was reduced following hybridization with CaTiO_3_-NPs, and this effect was dose-dependent. In contrast to pure SF and SF@CaTiO_3_-1, the results showed that the porous size of SF@CaTiO_3_-5 and SF@CaTiO_3_-10 was less than 60%. According to the evaluation, the CaTiO_3_-NPs were indeed nanosized, and it was found that certain nanoparticles could congregate in the same spot to form a cluster. Furthermore, it was shown in prior work that the porous size of pure SF scaffolds was around 80 μm, which is significantly greater than the diameter of CaTiO_3_-NPs. Consequently, it was plausible to assume that nanoparticles filling the pores of the SF scaffold was mostly responsible for the porosity reduction of the scaffold.

The porous size and distribution of CaTiO_3_-NPs within the fabricated SF@CaTiO_3_ hybrid scaffolds were identified to illuminate the scaffolds' hidden micro-morphology. All the scaffolds were found to have a blatantly porous structure, as seen in Fig. 4B. The red arrows show where nanoparticles were seen in the SF@CaTiO_3_ hybrid nanoparticles but not in the pure SF scaffolds' pores. Additionally, more nanoparticles were found with the enhanced CaTiO_3_ concentration, and some particle aggregation was found. The pore size data also showed that the pore width decreased with increasing CaTiO_3_ concentration, although there was no discernible difference between the scaffolds. Though the decreased porosity and pore size, the porous structure in the hybridized scaffolds could still provide suitable properties for cell ingrowth, nutrition exchange, and substance transportation [43].

**Fig. 5:**
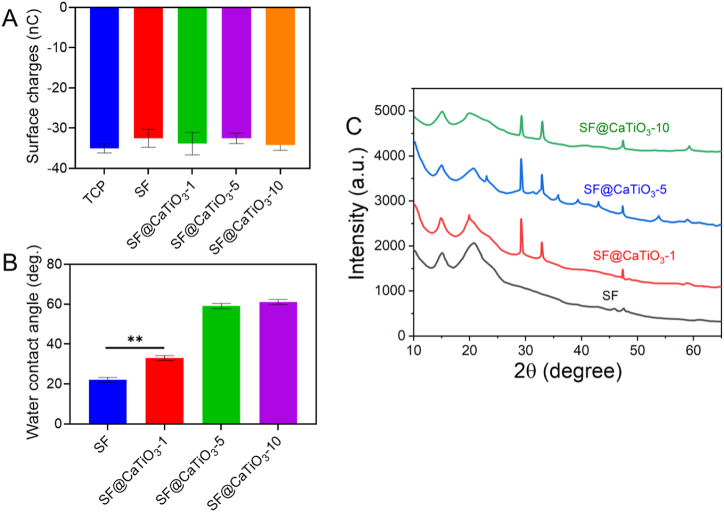
A) Surface charges on SF, SF@CaTiO_3_-1, SF@CaTiO_3_-5, and SF@CaTiO_3_-10. B) Water contact angle of pure SF, SF@CaTiO_3_-1, SF@CaTiO_3_-5, and SF@CaTiO_3_-10. C) X-ray diffractograms of the prepared pure SF, SF@CaTiO_3_-1, SF@CaTiO_3_-5, and SF@CaTiO_3_-10. Data are presented as mean ± SD. **P < 0.01.

Fig. 5A displays the surface charges of SF into CaTiO_3_, as determined by the TCP. The TCP was utilized as a baseline for comparison. TCP surface charges were around −36 nC, substantially lower than those of the other samples, whereas SF scaffolds had the highest surface charge values at about −30.6 nC. After CaTiO_3_ hybridization, the surface charge of the SF was considerably reduced to −33 nC and −35 nC. These findings demonstrated unequivocally that the CaTiO_3_-NPs reduced surface charge and rendered the surface more negatively charged. Because of its abundance of protonated amino groups, Silk fibroin is known to carry positive charges. The surface charge was reduced by the hybridized CaTiO_3_-NPs, which may mitigate the impact of amino group protonation. Unfortunately, the specific mechanism was not established and will require more research.

The water contact angle (WCA) was evaluated using DH_2_O at various sites on different scaffolds to investigate the effect of CaTiO_3_ on the scaffold's wettability. Fig. 5B shows the CA of DH2O on pure SF, SF@CaTiO3-1, SF@CaTiO3-5, and SF@CaTiO3-10. The contact angle of the pure SF scaffolds was roughly 19°, much less than the contact angles of the other samples, suggesting a hydrophilic surface of the scaffolds. Next, CaTiO_3,_ the WCA, grew to 31° in SF@CaTiO_3_-1 and around 56° in SF@CaTiO_3_-5 and SF@CaTiO_3_-10. Consequently, the wettability of pure SF was changed by adding CaTiO3, and the hydrophobicity was much enhanced compared to pure SF and SF@CaTiO_3_-1. The surface's physical and chemical characteristics play a significant role in determining a biomaterial's wettability. CaTiO_3_-NPs may vary in wettability due to differences in porosity, shape, analysis of hydrophilic functional groups (-OH, –NH_2_ carried in Silk fibroin), and surface charges [45,46].

The existence of diffraction peaks can be used to assess the material's mechanical order at an extended range or periodicity. Fig. 5C displays the XRD crystal structure analysis of the SF@CaTiO_3_ hybrid scaffolds. As a point of comparison, pure Sf scaffolds were utilized. Fig. 5C reveals that the diffraction pattern of pure SF scaffolds mostly revealed an amorphous solid morphology with the low intensity of two prominent reflections in 2ɵ at 15.2° and 20.0°. This finding was in good agreement with that previously published investigation. The XRD pattern showed that the Silk fibroin has low crystallinity due to its wide characteristic peaks. Compared with pure SF scaffolds, the XRD patterns of hybridized SF scaffolds with CaTiO_3_ (Fig. 5C) were noticeably different. The three SF@CaTiO_3_ hybrid scaffolds did not appear to be significantly different from one another. The hybridized scaffolds displayed the typical peaks of CaTiO_3_ at 30.1°, 32.9°, 48.2°, and 58.8°, showing the high degree of crystallinity of CaTiO_3_.

### 3.2 Proliferation of Schwann cells

**Fig. 6:**
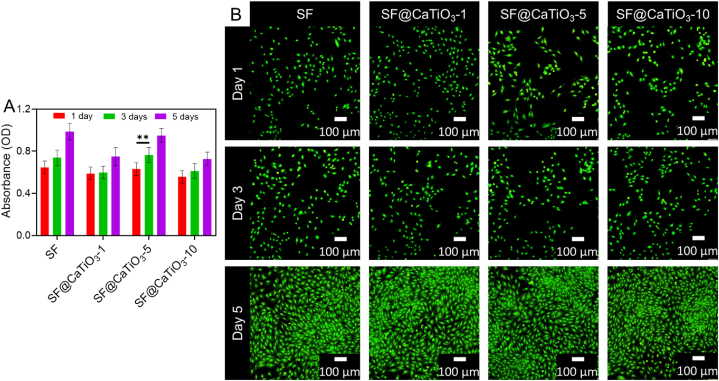
A) Proliferation of Schwann cells in SF, SF@CaTiO_3_-1, SF@CaTiO_3_-5, and SF@CaTiO_3_-10 scaffolds for 1, 3, and 5 days. B) The fluorescence microscopy examined the proliferation of the cells. Data are presented as mean ± SD. Scale bar 100 μm **P < 0.01.

Schwann cells are essential in the peripheral nervous system because of the function they play in facilitating regeneration and nerve repair. These cells might wrap the axon in myelinated sheaths and secrete growth factors to stimulate regeneration [47,48]. To this end, Schwann cells have traditionally been utilized as cells to examine peripheral nerve regeneration in vitro. Although research on the impact of osteoblastic cells with CaTiO_3_ for bone tissue engineering already exists, the impact of this material on Schwann cells has not been previously investigated. Early on, the CCK-8 assay was used to assess Schwann cell adhesion and proliferation in this work. In Fig. 6A, Schwann cells are shown proliferating clearly on pure SF and CaTiO_3_ from different days of the cell culture. The highest absorbance was found on day 5, suggesting the greatest number of scaffold cells. However, the quantity of Schwann cells at the same period differed across all scaffolds. The cell growth pattern from day 1 to day 5 of culture was consistent with the following order: SF@CaTiO_3_-5 > SF > SF@CaTiO_3_-1 > SF@CaTiO_3_-10. SF@CaTiO_3_-5 hybrid scaffolds yielded the highest absorbance, whereas SF@CaTiO_3_-10 scaffolds yielded the lowest. It's also worth noting that increasing the CaTiO_3_ concentration didn't influence Schwann cell growth noticeably (Fig. 6B). Thus, future Schwann cell proliferation and nerve regeneration may be further influenced by the variation in released Ca^2+^ ions. Findings suggested that optimal CaTiO_3_ hybridization concentrations might stimulate Schwann cell growth.

### 3.3 Distribution and morphology of schwann cells

**Fig. 7:**
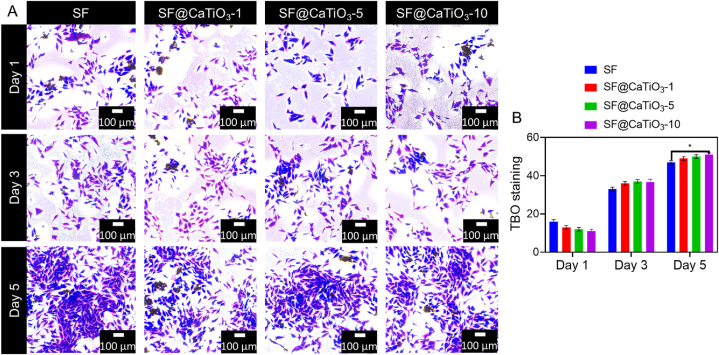
A) Examination of Schwann cells in pure SF, SF@CaTiO_3_-1, SF@CaTiO_3_-5, and SF@CaTiO_3_-10 hybrid scaffolds via delivery by TBO staining. B) Quantification ratio of TBO staining. Data are presented as mean ± SD. Scale bar 100 μm *P < 0.05.

TBO staining was used to assess the location and shape of Schwann cells in different fabricated scaffolds. Since TBO is a cationic dye, it stains the negative charged cells, leaving the positively charged Silk fibroin unaffected. In addition, we have previously found that TBO may be utilized to dye Schwann cells blue effectively. Fig. 7A demonstrates that, except for the pure SF, Schwann cells had no aggregation characteristics as they were uniformly dispersed across the scaffolds. Similar patterns were seen for cell proliferation, with fewer cells present on day 1 of culture for all scaffolds compared to days 3 and 5. In addition, Schwann cells in SF@CaTiO_3_-5 hybrid scaffolds showed clear interconnection networks at day 5, which were not present in the other scaffolds. It was hypothesized that improved cell-to-cell communication would aid cell migration, proliferation, and the transmission of signals. As a result, it may be possible to speed up the process of peripheral nerve regeneration. In addition, it is worth noting that while some cells entered the pore structure of the hybrid scaffolds throughout the culture days, they were not always easily detected by an optical microscope. These findings showed that Schwann cells might be dispersed uniformly across the SF@CaTiO_3_ hybrid scaffolds. In addition, the cell viability was examined by fluorescence microscopy images, which revealed the percentage of cell viability on different days after the treatment with different samples (Fig. 7B).

The findings further suggested that hybridized CaTiO_3_-NPs had a significant role in controlling Schwann cell shape. Attachment and proliferation of the MC3T3-E1 preosteoblast cell varied with the concentration of the two drugs. The closeness in composition between HAp/CaTiO_3_ scaffolds and bone tissue in nature was also cited as the rationale for their success in fostering the regeneration of bone tissue. Therefore, incorporating CaTiO_3_-NPs into Silk fibroin may have aided in the final component, structural, or biological cue match between the natural neural tissue and hybrid scaffolds in the current investigation. But the precise process is still unknown; therefore, further research is needed to validate the phenomena.

### 3.4 Analysis of cell protrusion

**Fig. 8:**
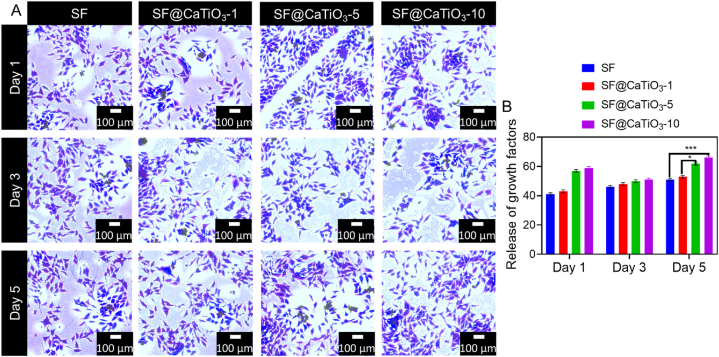
A) Release of growth factors in Schwann cells in SF, SF@CaTiO_3_-1, SF@CaTiO_3_-5, and SF@CaTiO_3_-10. B) Quantification ratio of release of growth factors. Data are presented as mean ± SD. Scale bar 100 μm ***P < 0.001.

**Fig. 9:**
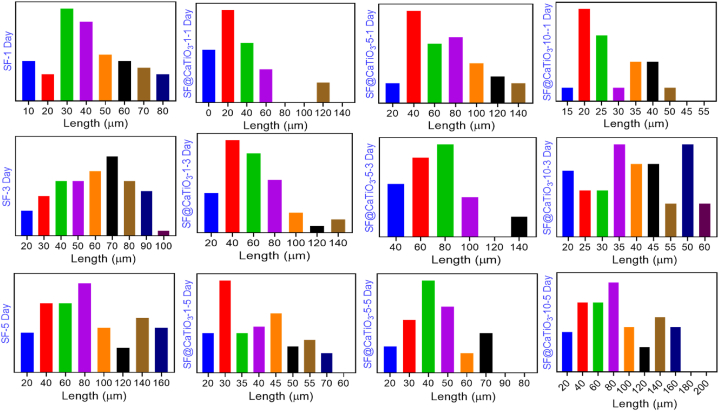
Release of growth factors in Schwann cells in SF, SF@CaTiO_3_-1, SF@CaTiO_3_-5, and SF@CaTiO_3_-10 for different days (1,3 and 5).

Schwann cells were often spindle-shaped with 2 or 3 protrusions, which may have been an indicator of their bioactivity. Therefore, the study further described how Schwann cells proliferate and spread out in different scaffolds. TBO staining was examined by Schwann cells with higher magnification. It was used to evaluate the impact of CaTiO_3_ scaffolds on morphological differences by analyzing the length and number of cell protrusions (Fig. 8A). The cells' protrusion quantity and length varied dramatically between scaffold types. Schwann cells showed greater and slower cell projections than the rest of the scaffold types when cultured on pure SF scaffolds or SF@CaTiO_3_-5 hybrid scaffolds. Fig. 8B displays the average amount of cell protrusions by cells. On day 1, cells in scaffold types prolonged one or two protrusions, but only a small percentage of cells in SF and SF@CaTiO_3_-5 revealed three or more protrusions. SF, SF@CaTiO_3_-1 and SF@CaTiO_3_-5, and SF@CaTiO_3_-5 displayed two or three cell protrusions after 3 and 5 days of culture, respectively. More than 80% of Schwann cells in SF@CaTiO_3_-5 had two or three-cell protrusions, a far greater rate than in other scaffolds. CaTiO_3_ hybridization positively stimulates cell protrusions outgrowth at an effective concentration. Furthermore, the length of protrusions extending from the soma of Schwann cells was measured and depicted in Fig. 9. In SF@CaTiO_3_-5 scaffolds, the protrusion length was 40–120 μm on day 1 of culture, while it was less than 50 μm in most other types of scaffolds. There was a general increase in the length of Schwann cell protrusions on day 3 across all scaffold types. The SF@CaTiO_3_-5 scaffolds had the longest cell protrusions, measuring 140 μm in length, whereas the SF@CaTiO_3_-10 scaffolds had the shortest, measuring less than 70 μm. Schwann cell protrusion did not lengthen after 5 days in culture in any of the scaffolds, although it was significantly shorter in the SF@CaTiO_3_-5 scaffolds compared to those produced on days 1 and 3. Cell proliferation as measured by cell counting (CCK-8) analysis and cell distributions revealed by Toluidine blue stain (TBO) indicated that the highest cell density was seen on day 5 of culture in the SF@CaTiO_3_-5 scaffolds. Schwann cell protrusion length was reduced, suggesting that the cell-cell contact inhibitory effect has a detrimental impact on elongation. The findings confirmed that Schwann cell outgrowth was enhanced following CaTiO_3_-NPs hybridization with SF scaffolds, suggesting that this approach might be useful for treating peripheral nerve regeneration.

### 3.5 Biological function assessment

**Fig. 10:**
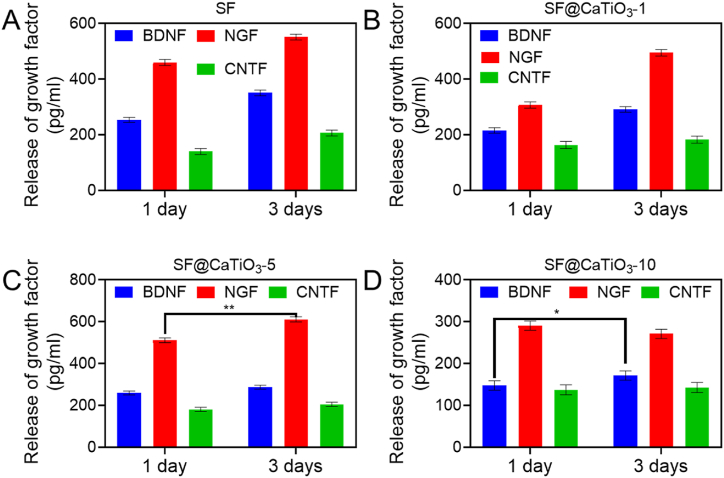
Assessment of protrusion outgrowth of Schwann cells. Histograms display the release of BDNF, NGF, and CNTF by Schwann cells cultured for 1 and 3 days as measured by ELISA assay. Data are presented as mean ± SD. *P < 0.05, **P < 0.01.

Schwann cells facilitate neuron regeneration by performing their regular biological activity. Release amounts of BDNF, CNTF, and NGF from cells after 1 and 3 days of culture were examined to analyze the biological activity of the cells in SF@CaTiO_3_-1, SF@CaTiO_3_-5, and SF@CaTiO_3_-10. Schwann cells cultured on SF@CaTiO_3_-10 scaffolds secreted considerably less NGF, CNTF, and BDNF across all time points (Fig. 10). With prolonged incubation, Schwann cells released more of all three biofactors from the various scaffolds. At each time, cellular NGF release in SF@CaTiO3-5 scaffolds was also considerably greater than in other scaffold types. However, when comparing the amount of BDNF and CNTF released, there was no discernible change among SF@CaTiO_3_-5 and the rest of the scaffolds. Several growth factors aid axon development and elongation during peripheral nerve regeneration. This study's results showed that using SF@CaTiO_3_ hybrid scaffolds increased the growth release factors from cells with no significantly compromising cell bio function when using lower quantities of CaTiO_3_-NPs. However, the release of many growth factors was inhibited at higher quantities. It was expected that CaTiO_3_-NPs would stimulate Schwann cells, producing numerous growth factors that would aid in the effective regeneration of nerve fibers. As a result, it is sufficient to expect that the findings may deliver a crucial investigational origin for developing synthetic hybrid scaffolds for different kinds of nerve regeneration that do not interfere with normal physiologic cell activity. In addition to the impacts of SF@CaTiO_3_ scaffolds on cells' shape, proliferation, function, etc., it is crucial to get a deeper understanding of the cellular responses at the molecular level, such as signal pathways, protein variation, and gene expression analysis.

## 4 Conclusion

The current study entails the construction of porous SF@CaTiO_3_ scaffolds and an evaluation of their practicality, scaffold characteristics, and biocompatibility in the context of peripheral nerve regeneration. Successful hybridization of CaTiO_3_-NPs onto scaffolds led to changes in the shape, wettability, surface charge, and porosity of the different formulations of SF@CaTiO_3_. In vitro evaluation results reveal that the porous SF@CaTiO_3_ increases cell attachments, cell proliferations, and cell biological function preservations, suggesting possibly enormous relevance for synthetic peripheral nerve implants in humans. This research might pave the way for better biomedical device design for various tissue engineering uses and provide a deeper insight into the impact of hybridized biomaterial scaffolds on peripheral nerve regeneration.

## Author contribution statement

Jincheng Zhang, Yiwen Xu: Conceived and designed the experiments; Contributed reagents, materials, analysis tools or data; Wrote the paper.

Yingqi Zhang: Analyzed and interpreted the data; Contributed reagents, materials, analysis tools or data.

Lei Chen, Yeqing Sun: Analyzed and interpreted the data.

Jia Liu: Contributed reagents, materials, analysis tools or data.

Zhitao Rao: Analyzed and interpreted the data; Wrote the paper.

## Funding statement

This research did not receive any specific grant from funding agencies in the public, commercial, or not-for-profit sectors.

## Data availability statement

Data will be made available on request.

## Declaration of interest’s statement

The authors declare no competing interests.
